# “It’s so Cute I Could Crush It!”: Understanding Neural Mechanisms of Cute Aggression

**DOI:** 10.3389/fnbeh.2018.00300

**Published:** 2018-12-04

**Authors:** Katherine K. M. Stavropoulos, Laura A. Alba

**Affiliations:** Graduate School of Education, University of California, Riverside, Riverside, CA, United States

**Keywords:** event-related potentials, cute aggression, reward, emotion, RewP, N200

## Abstract

The urge people get to squeeze or bite cute things, albeit without desire to cause harm, is known as “cute aggression.” Using electrophysiology (ERP), we measured components related to emotional salience and reward processing. Participants aged 18–40 years (*n* = 54) saw four sets of images: cute babies, less cute babies, cute (baby) animals, and less cute (adult) animals. On measures of cute aggression, feeling overwhelmed by positive emotions, approachability, appraisal of cuteness, and feelings of caretaking, participants rated more cute animals significantly higher than less cute animals. There were significant correlations between participants’ self-report of behaviors related to cute aggression and ratings of cute aggression in the current study.

**N200:** A significant effect of “cuteness” was observed for animals such that a larger N200 was elicited after more versus less cute animals. A significant correlation between N200 amplitude and the tendency to express positive emotions in a dimorphous manner (e.g., crying when happy) was observed.

**RewP:** For animals and babies separately, we subtracted the less cute condition from the more cute condition. A significant correlation was observed between RewP amplitude to cute animals and ratings of cute aggression toward cute animals. RewP amplitude was used in mediation models.

**Mediation Models:** Using PROCESS (Hayes, 2018), mediation models were run. For both animals and babies, the relationship between appraisal and cute aggression was significantly mediated by feeling overwhelmed. For cute animals, the relationship between N200 amplitude and cute aggression was significantly mediated by feeling overwhelmed. For cute animals, there was significant serial mediation for RewP amplitude through caretaking, to feeling overwhelmed, to cute aggression, and RewP amplitude through appraisal, to feeling overwhelmed, to cute aggression. Our results indicate that feelings of cute aggression relate to feeling overwhelmed and feelings of caretaking. In terms of neural mechanisms, cute aggression is related to both reward processing and emotional salience.

## Introduction

Cute aggression is defined as the urge some people get to squeeze, crush, or bite cute things, albeit without any desire to cause harm. Aragón et al. (2015) initially operationalized the phenomenon of “cute aggression” through individual self-reports while viewing cute stimuli. The authors investigated cute aggression using pictures of baby humans and animals via an online survey. Findings indicated that for infantile babies (e.g., images that had been altered to have large eyes and chubby cheeks; Sherman et al., 2013) and baby animals, there was a relationship between being overwhelmed by positive feelings and the expression of cute aggression (Aragón et al., 2015).

Cute aggression has been discussed as an example of dimorphous expression of emotions. Dimorphous expression refers to someone experiencing a strong emotion of one type (e.g., happy or sad) but expressing the opposite emotion. For example, some people report laughing when they are sad, or crying when they are happy. Typically, expressions of emotions are broad, such as smiling when happy or frowning when sad (Aragón, 2017; Aragón and Bargh, 2018). However, the emotions we express to very cute stimuli are complex overlapping emotions that communicate one category of emotion (Aragón, 2016). Most of the feelings for cute aggression can be viewed as contradictory, such as in the event of receiving a new puppy and simultaneously crying and smiling.

Authors hypothesize that “cute aggression” may serve as a bottom-up mechanism for regulating overwhelming positive emotions. In support of this hypothesis, Aragón et al. (2015) found that the relationship between ratings of how cute something is, and cute aggression was mediated by the experience of being overwhelmed by positive feelings. The authors posited that evolutionarily, it would not have been adaptive to become incapacitated by positive feelings in response to a very cute baby who required caretaking. Therefore, the dimorphous expression of cute aggression may occur to regulate these overwhelmingly positive emotions (Aragón et al., 2015). Further evidence for this was observed in the relationship between appraisal of cuteness (e.g., how cute something is), expressions of caretaking, and feeling overwhelmed. Behavioral data suggest that appraisal and expressions of caretaking are mediated by being overwhelmed, and that feelings of cute aggression and caretaking are highly correlated (Aragón et al., 2015).

Responding to the cuteness of an animal or baby is not a new phenomenon. From an evolutionary perspective, a human’s ability to respond to the cuteness of an infant or animal triggers innate processes for caregiving, known as the baby schema (Lorenz, 1943; Lorenz and Martin, 1971; Hildebrandt and Fitzgerald, 1979). Many studies have shown that viewing images of babies with round faces and high foreheads were perceived as cute and elicited a higher response for caretaking when compared to babies with narrow faces and low foreheads (Glocker et al., 2009a,b). Moreover, the appearance of cuteness has also elicited caretaking behaviors among adults, even before becoming parents (Volk and Quinsey, 2002; Esposito et al., 2014). For instance, in a study using a hypothetical adoption scenario, findings suggest that cuteness and health of the child was the primary reason for adopting a child when compared to physical resemblance and happiness level among women (Volk and Quinsey, 2002). Although cuteness was the primary reason for women to adopt a child, men in the study reported physical resemblance as the primary reason, followed by cuteness.

Cuteness has also been shown to elicit social engagement, suggesting that humans may assess the value of sociability in children (Sherman and Haidt, 2011). For instance, more affection and playfulness were shown among mothers of cuter infants when compared to mothers with less cute infants (Langlois et al., 1995). Indeed, Sherman and Haidt (2011) suggested that cuteness evoked social behaviors that are similar to caretaking (e.g., touching, holding) which provides evidence for the indirect association of social engagement and caretaking behavior. Moreover, assessing the value of sociability in a child may provide evidence as to why non-parents report caretaking behaviors when viewing cute infants (Sherman and Haidt, 2011).

Interestingly, previous research indicates that the concept of a baby schema triggering a “cute response” extends to animals (e.g., Archer and Monton, 2011; Little, 2012). Behavioral evidence suggests that both children and adults rated highly infantile images of babies, puppies, and kittens as more cute than the less infantile versions of all three species (Borgi et al., 2014). Further, this effect was particularly pronounced for pet owners, which suggests that familiarity with common household pets (e.g., dogs and cats) is important. Another study measured brain activity in mothers while viewing images of their own child vs. an unfamiliar child, as well as while viewing images of their own dog vs. an unfamiliar dog. The authors found increased activation in brain areas related to reward, social cognition, and affiliation in response to both familiar children and dogs (e.g., own child, own dog) compared to unfamiliar children and dogs (Stoeckel et al., 2014). Although not directly related to “cuteness” findings from Stoeckel et al. (2014) suggest the importance of familiarity when measuring reward-related brain activity when viewing both humans and animals. Taken together, these studies provide evidence that the concept of a baby schema extends to animals, and is not specific to human babies. It is important to note, however, that these studies did not explore behavioral ratings or brain activity related to cute aggression, but rather measured behavioral responses of cuteness and brain activity in regions related to reward and social affiliation.

Although no previous studies (to our knowledge) have investigated the neural underpinnings of cute aggression, previous research has been conducted on the neural reward response to more versus less cute babies in nulliparous women (Glocker et al., 2009b). The authors found that more cute babies (images manipulated in accordance with Lorenz’ ‘baby schema’) elicited increased activity in the nucleus accumbens, which is a critical structure in the mesocorticolimbic reward system.

The current study was designed to identify and measure neural underpinnings of cute aggression. Neural correlates for emotional salience and reward may provide insight into this phenomenon. Using electrophysiology, specifically event-related potentials (ERPs), the current study measured neural components related to emotions (N200), reward anticipation (SPN), and reward processing (RewP).

### N200

The N200 is a negative ERP component peaking 200–300 ms post stimulus (Squires et al., 1976, 1977). Numerous studies have shown that the N200 is related to the emotional content of stimuli (Balconi and Lucchiari, 2005; Balconi and Pozzoli, 2009; Kanske and Kotz, 2010, 2011). Kanske and Kotz (2011) used an emotional valence flanker task where participants responded to the print color of the target word where it was either neutral or emotional and found a difference in N200 amplitude for emotional versus neutral trials. Similar findings for the N200 have been observed for facial expressions, with larger amplitude N200s observed after emotional faces compared to neutral faces (Streit et al., 2000; Herrmann et al., 2002; Balconi and Pozzoli, 2003). Taken together, these findings have identified the N200 as a neural correlate of emotional significance. Given that cute aggression is hypothesized to be a response to strong positive emotions, the N200 is a plausible target when exploring neural correlates of cute aggression.

### SPN

Another way to examine neural correlates of cute aggression is to explore reward-related ERP components. The stimulus preceding negativity (SPN) is a slow wave component that reflects the expectation of reward stimuli (Damen and Brunia, 1987). The significance of the SPN is typically conceptualized as emotional anticipation (Chwilla and Brunia, 1991; Kotani et al., 2001, 2003), and is thought to reflect activity in the insula (Kotani et al., 2009, 2015). The SPN is typically measured after participants make a motor response and before feedback onset in a decision-making task (Brunia et al., 2012). The SPN is sensitive to reward magnitude and is consistently larger in reward versus no-reward conditions (Kotani et al., 2001, 2003; Ohgami et al., 2004). Though the SPN is typically measured in decision-making tasks, previous research has reported that the SPN can be observed when anticipating affective upcoming stimuli without a task (Takeuchi et al., 2005; Poli et al., 2007; Parker and Gilbert, 2008) For instance, Poli et al. (2007) used a S1–S2 paradigm in which the content of the forthcoming emotional pictures (S2) could be predicted by S1. Findings indicated that there was a larger SPN when anticipating strongly affective pictures when compared to neutral pictures. Given the relevance of the SPN to reward and affective anticipation, we identified this component as potentially relevant to cute aggression. To our knowledge, no research has evaluated the association between the SPN, cute stimuli, and expressions of aggression.

### RewP

The RewP response is a positive component that peaks 300 ms after rewarding stimuli (Miltner et al., 1997). Numerous studies have shown that the RewP is elicited by positive feedback (Baker and Holroyd, 2011; Foti et al., 2011) and suppressed by negative feedback (Feedback Negativity; FN; Bress et al., 2012) for both adults and adolescents. Previous research on losses and gains has suggests that the RewP was larger when associated with actual rewards and smaller for non-rewards (Carlson et al., 2011; Bress and Hajcak, 2013). The RewP predicted individual differences among sensitivity levels for rewards when evaluated using both behavioral and self-report measures (Bress and Hajcak, 2013). For instance, among college students, higher scores on the Reward Responsiveness Scale (RRS; Van den Berg et al., 2010) were correlated with a heightened RewP response on a bias reward detection task (Pizzagalli et al., 2005), suggesting an increased interest in rewarding feedback (Bress and Hajcak, 2013). Overall, findings suggest that the RewP is a neural correlate of positive and negative reward stimuli (for a review, see Proudfit, 2015). To date, no research has investigated the relationship between cute images and the RewP.

### Current Study

This study aimed to extend the behavioral findings of Aragón et al. (2015) by examining the neural correlates of cute aggression. We are unaware of any study to date that has measured brain activity related to cute aggression or related brain activity to participants’ report. We hypothesized that amplitude of the N200 when viewing “more cute” pictures would relate to expressions of cute aggression either in mediation models or simple correlations. A second potential mechanism for cute aggression relates to reward anticipation and processing. We hypothesized that expressions of cute aggression might be related to finding stimuli particularly rewarding. As the current study involved passive viewing (rather than an overt task), we hypothesized that expressions of cute aggression might relate to SPN amplitude, Reward positivity (RewP) amplitude, or both. We also measured whether individuals’ self-reports about actions related to cute aggression were correlated with behavioral ratings of cute aggression in the current study. Finally, we explored the relationship between brain and behavioral ratings using both correlations and mediation models (e.g., whether the relationship between N200 amplitude and cute aggression is mediated by feeling overwhelmed).

## Materials and Methods

### Participants

We tested 54 protect adult participants (20 males and 34 females) between 18 and 40 years old (*M* = 20.05, *SD* = 3.33). Participants had no history of developmental disabilities or psychiatric conditions and were not taking any medications for psychiatric or neurological conditions (as per self-report). One participant was tested but later excluded because we learned she had a previous psychiatric diagnosis (which was unknown at the time of testing). Participants were recruited through the University of California, Riverside subject pool and from on-campus flyers. All participants were over 18 years of age and signed a consent form. All procedures were approved by the University of California, Riverside Institutional Review Board (IRB).

### Stimuli and Task

#### Stimuli

The current study had four blocks of trials, each containing different images: more cute (baby) animals, less cute (adult) animals, more cute babies, less cute babies. Stimuli in the baby and adult animal conditions were the same as reported and validated by Aragón et al. (2015). Aragón et al. (2015) searched online for animals with infantile features (e.g., large eyes, large head), and for animals who were older and lacked those characteristics. They identified and validated eight infantile images, and eight images of older animals. The eight images were of the following animal species: elephant, duck, pig, cat, monkey, dog, and rabbit. We obtained the photographs from the authors and used them in the “more cute animals” and “less cute animal” conditions, respectively.

Stimuli in the more and less cute baby conditions were the same as reported by Aragón et al. (2015), obtained with permission from the research group who created and validated the stimuli (Sherman et al., 2013). The eight photographs of infants (two female, six male) were morphed such that in the “more cute” condition, the infants had more infantile characteristics (e.g., larger eyes, fuller cheeks), and in the “less cute” condition they had less infantile characteristics (e.g., smaller eyes, less full cheeks). Note that the subjects of the photographs were the same in both the “more cute baby” and “less cute baby” conditions—the variation between them was in the morphing of the photographs. These infant photographs in each condition were originally validated in the Sherman et al. (2013) study, and validated independently by Aragón et al. (2015).

#### Task

Participants did not engage in any overt task. Each participant saw all four blocks of stimuli (more cute animals, less cute animals, more cute babies, less cute babies) in a random order. Randomization was done using a random number generator prior to each subject. Within each block, each photograph was shown four times (for a total of 32 trials in each block) in a pseudo-random order such that no photograph was repeated more than twice in a row. Participants were told that they would be passively viewing different photographs on the screen, and that they would be filling out questionnaires about each set (block) of photographs.

Between each block, participants were asked to complete behavioral measures indicating how they felt about the pictures they saw. We note that due to the design of the current study, participants were asked to respond to behavioral measures after each block, and to answer each question about “how they felt about the photographs they just saw.” Thus, participants were not making ratings about each individual photograph, but were rating how they felt in general about each *category* of picture (e.g., we did not obtain ratings about how participants felt about *each* of the eight images of baby animals separately, but rather, about how they felt about *all* of the baby animal photographs). Including time for behavioral ratings between each block, the total duration of the EEG portion of the experiment was approximately 25 min. After the EEG portion of the experiment, participants filled out an additional questionnaire (see the section “Behavioral Measures”).

### Behavioral Measures

Prior to beginning the EEG portion of the study, participants completed a questionnaire related to dimorphous expressions of emotions (used with permission from Aragón et al., 2015). We were particularly interested in participants’ dimorphous expressions of positive emotions, which was measured by taking the average of the following three items (rated on a scale of 1–6): (1) ‘I cry while watching the happiest of moments of movies,’ (2) ‘When I am feeling strong positive emotions, I express with negative expressions,’ and (3) ‘When I am feeling a strong positive emotion (for example, extreme happiness, strong self of relief, strong feeling of connection to others etc.), my expression can look like I am feeling a negative emotion (for example I might cry, or scream as though in fear even though I am happy or excited’).

Between each block of EEG stimuli, participants were asked to fill out rating scales related to their feelings about each block of pictures. Rating scales were the same as reported in previous research on cute aggression, slightly modified to be completed in person rather than online (Aragón et al., 2015). At the top of the rating scale, the following statement was used to help participants understand the meaning and interpretation of the questions:

We ask about “playful aggression.” Playful aggression is in reference to the expressions that people show sometimes when interacting with babies (animals or people). Sometimes we say things and appear to be more angry than happy, even though we are happy. For example, some people grit their teeth, clench their hands, pinch cheeks, or say things like “I want to eat you up!” It would be difficult to ask about every possible behavior of playful aggression, so we ask generally about things of this kind—calling them playful aggressions.

Participants were shown statements and asked to rate how much they agreed with each one on a scale from 1 to 10, with 1–2 representing, “not at all true,” 3–4 representing “a little bit true,” 5–6 representing “true,” 7–8 representing “very true,” and 9–10 representing “completely true.” To capture the feeling of *cute aggression*, the average of the following statements was calculated: ‘I want to say something like, “grrr,”’ ‘I want to squeeze something,’ ‘I feel like pinching those cheeks!,’ ‘Saying ‘I want to eat you up!’ through gritted teeth,’ ‘Being playfully aggressive!’ To capture feelings of being *overwhelmed by emotion*, the average of the following statements was calculated: ‘I can’t handle it!,’ ‘I can’t stand it!,’ ‘I feel overwhelmed with positive feelings when I see these photographs.’ To investigate how much participants wanted to approach the subjects in the photograph (i.e., *approachability*), we used the item, ‘I want to approach the subjects in the photographs.’ For a rating of how cute participants thought images were, henceforth referred to as *appraisal*, we used the item ‘That’s cute!’ To measure *feelings of caretaking*, the average of the following statements was calculated: ‘I want to take care of it!,’ ‘I want to hold it!,’ ‘I want to protect it!’ See Table 1. It is important to note that because the words “playful aggression” were utilized in the behavioral measures, participants were not entirely “blind” to the conceptual framework of the current study. We utilized the above-referenced description for two reasons: (1) In order to keep methods consistent between the current study and that of Aragón et al. (2015), and (2) To provide context for participants to understand statements related to cute aggression. We wanted participants to understand that these expressions of aggression are made *in the absence of any intent to harm*. We thought that if participants believed the point of the research was to understand aggressive impulses made with the intent to harm the cute thing, we would not obtain accurate or representative responses.

**Table 1:** Behavioral ratings.

*Ratings*	*Items*
Cute aggression	(a) ‘I want to say something like, “grrr”’
	(b) ‘I want to squeeze something’
	(c) ‘I feel like pinching those cheeks!’
	(d) ‘Saying ‘I want to eat you up!’ through gritted teeth’
	(e) ‘Being playfully aggressive!’
Overwhelmed by emotion	(a) ‘I can’t handle it!’
	(b) ‘I can’t stand it!’
	(c) ‘I feel overwhelmed with positive feelings when I see these photographs’
Approachability	(a) ‘I want to approach the subjects in the photographs’
Appraisal	(a) ‘That’s cute!’
Feelings of caretaking	(a) ‘I want to take care of it!’
	(b) ‘I want to hold it!’
	(c) ‘I want to protect it!’


After the EEG protocol, participants filled out a “yes/no” questionnaire about things people say and do and were asked to indicate “yes” by checking all of the items that applied to them. In the section related to things people say, participants were asked if they had ever said any of the following: “it’s so cute I want to pinch it!,” “it’s so cute I want to squeeze it!,” “it’s so cute I want to bite it!” In the section related to things people do, participants were asked to indicate (1) if they had ever done this; (2) if you have wanted to do this but didn’t. The items were, “Pinched a cute animal?,” “Pinched a cute baby or child?,” “Squeezed a cute animal?,” “Squeezed a cute baby or child?,” “Bitten a cute animal?,” “Bitten a cute baby or child?”

### EEG Recording

EEG data were recorded using a Brain Products ActiCHamp system with electrodes located at 32 standard scalp locations of the extended international 10-20 system. Data was sampled at 500 Hz. Continuous EEG was amplified with a directly coupled high pass filter (DC), and a notch filter (60 Hz). Vertical and horizontal electrooculogram (EOG) were measured from electrodes located lateral to the outer canthus of each eye and from electrodes located above and below the left eye. All electrode impedances were kept below 50 kΩ. Offline, the EEG signals were re-referenced to the average of the two mastoid electrodes and filtered at 30 Hz and 0.01 Hz.

Each trial began with a fixation cross, which remained onscreen for 500 ms. Following the fixation cross, a 3,000 ms pause occurred (to allow the SPN to be measured). Following the pause, images of babies or animals were displayed for 1,000 ms. The inter-trial interval was varied randomly between 500 and 900 ms. Trials were time locked to the onset of the images of babies or animals. To measure reward anticipation, the baseline period was -3,300 to -3,100 ms, and the data was epoched from -3,300 to 100 ms. Similar to previous studies on the SPN component (Stavropoulos and Carver, 2013, 2014), mean amplitude was calculated from -210 to -10 ms from the following electrodes: F3/F4, C3/C4, P3/P4, and T7/T8. Note that in previous studies, electrodes T5 and T6 were used, and the current study utilized T7 and T8. This is due to differences in electrode placement and layout between electrode caps.

To measure emotion and reward processing, the baseline period was -200 to 0 ms, and the data were epoched from -200 to 800 ms. For the N200, based on previous studies (Kanske and Kotz, 2010, 2011), four regions of interests were defined: left anterior (FP1, F3, FC5), right anterior (FC6, F4, F8), left posterior: (CP5, P3, P7, O1) and right posterior (O2, P4, P8, CP6). Peak amplitude was detected within the following time window: 150–225 ms. For the RewP, based on previous studies (Oumeziane and Foti, 2016) mean amplitude was calculated for each condition as the average of frontocentral electrodes (Fz, FC1, FC2) between 250 and 350 ms. The RewP was defined as the difference between the more cute and less cute condition for animals and babies separately.

Trials containing electrophysiological artifacts were excluded from the averages. Artifacts were removed via a four-step process. Data were visually inspected for drift exceeding ±200 mV in all electrodes, high frequency noise visible in all electrodes larger than 100 mV, and flatlined data. Following inspection, data were epoched and eye blink artifacts were identified using independent component analysis (ICA). Individual components were inspected alongside epoched data, and blink components were removed. To remove additional artifacts, we utilized a moving window peak-to-peak procedure in ERPlab (Lopez-Calderon and Luck, 2014), with a 200 ms moving window, a 100 ms window step, and a 150 mV voltage threshold. Participants with less than 10 trials in any condition were excluded from statistical analysis. Our final analyses for reward anticipation (SPN) included 51 participants (two were excluded for having insufficient trials), and final analysis for emotion processing (N200) and reward processing (RewP) included 49 participants (four were excluded for having insufficient trials). Average number of accepted trials were calculated for each condition: Cute animals (*M* = 28.9, *SD* = 4.4), less cute animals (*M* = 29.7, *SD* = 3.1), more cute babies (*M* = 29.2, *SD* = 3.5), and less cute babies (*M* = 29.3, *SD* = 4.7). No significant differences were observed between the number of accepted trials between the more versus less cute conditions for either babies or animals (*ps* > 0.1).

### Data Analytic Plan

Paired *t*-tests were used to test for differences in behavioral ratings between conditions: more versus less cute animals and babies. For animals and babies separately, paired-sample *t*-tests were run on the following behavioral ratings: ratings of cuteness (appraisal), cute aggression, being overwhelmed, approach, and caretaking (Table 1). To explore participants’ behaviors related to cute aggression, we calculated how many participants responded affirmatively to each item of the “things people say and do” questionnaire and performed bivariate correlations with these behaviors and ratings of cute aggression in the current study (Table 2).

**Table 2:** Paired-sample *t*-tests for more versus less cute animals.

*Behavioral item*	*More cute: Mean rating (SD)*	*Less cute: Mean rating (SD)*	*t-value*	*p-value (2-tailed)*
Appraisal	8.23 (2.39)	5.56 (2.75)	7.63	*p* < 0.001
Aggression	3.25 (1.6)	2.32 (1.23)	4.32	*p* < 0.001
Overwhelmed	3.49 (2.12)	2.38 (1.28)	5.56	*p* < 0.001
Approach	7.40 (2.80)	5.34 (2.92)	6.27	*p* < 0.001
Caretaking	6.91 (2.62)	4.91 (2.61)	5.60	*p* < 0.001


ERP analysis were performed using EEGlab (Delorme and Makeig, 2004) and ERPlab (Lopez-Calderon and Luck, 2014). Statistical analyses were performed using IBM SPSS (version 24). For the N200 and SPN we used repeated-measures analysis of variance (ANOVA) to test for differences between conditions, hemispheres, and electrode locations. Greenhouse-Geisser corrected degrees of freedom are reported to account for violations of sphericity. The Reward positivity (RewP) is a difference wave calculated from a single electrode cluster by subtracting the less rewarding condition from the more rewarding condition. In the current study, it was calculated by subtracting the “less cute” condition from the “more cute” condition for babies and animals separately. Therefore, we did not run repeated measures ANOVAs to compare amplitude between conditions. The RewP was utilized in mediation models and correlations (reported below). To test for correlations between ERP components and behavioral ratings of cute aggression bivariate correlations were conducted in SPSS. Simple correlations between N200 and RewP amplitude and behavioral measures were run in SPSS.

To test for mediation effects, we used the SPSS PROCESS plug-in (Hayes, 2018). Relationships of interest were estimated using bootstrapping procedures, by resampling the data 5,000 times. Bootstrapping was also used to obtain confidence intervals for mediation effects. Two simple mediation models were tested using PROCESS Model 4 (Hayes, 2018): (1) We hypothesized that the relationship between appraisal (*X*) and expressions of cute aggression (*Y*) would be mediated by how overwhelmed participants felt (*M*_1_). (2) We hypothesized that the relationship between N200 amplitude (*X*) and expressions of cute aggression (*Y*) would be mediated by how overwhelmed participants felt (*M_1_*).

Additionally, we tested three serial mediation models using PROCESS Model 6 (Hayes, 2018): (1) To test whether the relationship between feelings of caretaking (*X*) and expressions of cute aggression (*Y*) would be serially mediated by appraisal (*M_1_*) and feelings of being overwhelmed (*M_2_*) and (2) To test whether the relationship between RewP amplitude (*X*) and expressions of cute aggression (*Y*) would be serially mediated by appraisal (*M_1_*) and feeling overwhelmed (*M_2_*). (3) To test whether the relationship between RewP amplitude (*X*) and expressions of cute aggression (*Y*) would be serially mediated by feelings of caretaking (*M_1_*) and feeling overwhelmed (*M_2_*).

## Results

### Behavioral Analyses

Participants rated baby animals significantly higher than adult animals on all five items (all *p*s < 0.01). Ratings for each item are shown for animals in Table 2. No significant differences were observed between ratings of more versus less infantile babies (all *p*s > 0.1). To measure whether differences between the “more cute” versus “less cute” conditions were observed between genders, difference scores were calculated by subtracting the ratings in the “less cute” condition from ratings in the “more cute” condition for animals and babies separately. Thus, each participant had two difference scores for each of the five items reported in Table 2 (e.g., one for babies, and one for animals). Independent samples *t*-tests were run on the difference scores between genders. No significant differences were observed in either the animal or baby conditions for any of the behavioral rating items. Therefore, Table 2 shows data from all participants. For all subsequent analyses reported in the manuscript, behavioral ratings of “more cute” and “less cute” babies were collapsed due to lack of significant differences in behavioral ratings between conditions.

To explore what percentage of participants had said or done things related to cute aggression, frequencies were calculated for the 3 “things people say” items, and the 6 “things people do” items. Percentages of participants who responded affirmatively can be found in Table 3. To check for differences between males and females on endorsing these items, Chi-Square tests were run on each of the items with gender as a between subjects variable. No significant differences were observed between genders for how likely individuals were to endorse these items (all *p*s > 0.1) Thus, correlations reported below were not separated by gender.

**Table 3:** Percentage of participants responding “yes” to things people say and do, and correlations with ratings of cute aggression.

Things people say	Percent of participants who responded “yes”	Correlations with cute aggression (*p*-value)
“It’s so cute I want to pinch it!”	46%	Ns
“It’s so cute I want to squeeze it!”	64%	0.037 (for animals)
“It’s so cute I want to bite it!”	28%	0.008 (for animals)
**Things people do**	**Percent of participants who responded “yes”**	
Pinched a cute animal	28%	Ns
Pinched a cute baby/child	56%	Ns
Squeezed a cute animal	74%	0.004
Squeezed a cute baby/child	60%	0.045
Bitten a cute animal	16%	N/A
Bitten a cute baby/child	12%	N/A
**Want to do but have not done**	**Percent of participants who responded “yes”**	
Pinched a cute animal	6%	N/A
Pinched a cute baby/child	4%	N/A
Squeezed a cute animal	10%	N/A
Squeezed a cute baby/child	12%	N/A
Bitten a cute animal	6%	N/A
Bitten a cute baby/child	2%	N/A


To measure the relationship between ratings of cute aggression and items from the “things people say” and “things people do” questionnaire, bivariate correlations were run with “things people say” and “things people do” as categorical variables, and ratings of cute aggression as continuous. Items with less than 10 people responding “yes” or “no” (all items related to things people wanted to do but didn’t, having bitten an animal, and having bitten a cute baby or child) were not included in the bivariate correlations. See Table 3 for Pearson correlation values (note that correlations which were not run–due to having less than 10 people endorse the statement–are demarcated with “N/A”).

Note that because five correlations were run for each condition (e.g., five for babies and five for animals), Bonferroni corrections would change the threshold for significance to 0.01. Therefore, *p*-values that are below 0.05 but less than 0.01 are noted as “marginal,” whereas those under 0.01 are considered “significant.” A marginally significant relationship was observed between expressions of cute aggression for more cute animals and individual reports of ever having said, “it’s so cute I want to squeeze it!” (*p* = 0.037). Significant correlations were observed between ratings of cute aggression and reports of ever having said, “it’s so cute I want to bite it!” (*p* = 0.008), and between cute aggression and reports of ever having squeezed a cute animal (*p* = 0.004), such that individuals who reported having said or done these things reported significantly higher feelings of cute aggression than those who did not report having done or said them.

For babies, a marginally significant relationship was observed between cute aggression and individual reports of ever having squeezed a cute baby (*p* = 0.045). Individuals who reported having squeezed a cute baby exhibited significantly higher ratings of cute aggression in response to more cute babies compared to individuals who reported never having squeezed a cute baby.

### ERP

#### SPN

2 (cuteness) × 2 (hemisphere) × 4 (electrode position) repeated measures ANOVAs were run on animal and babies separately. No main effects or interactions of interest were observed (*p* > 0.05). An interaction between hemisphere and electrode was observed for animals, but this was not explored further as it was not related to “cuteness,” nor was it a main effect that would lead to re-analysis (based on collapsing across conditions). Though we hypothesized that the images used in the current study would be emotionally salient enough to elicit an SPN, we did not employ a traditional “response → feedback” or “S1, S2” paradigm. To confirm that brain activity in the 200ms prior to stimulus reflected a reliable SPN, four one-sample *t*-tests were run against 0 using in all four conditions, collapsed across hemisphere and electrodes. Results indicated that the average amplitude was not significantly different from zero in any of the four conditions (all *p*s > 0.05). Therefore, because we were unable to confirm that the SPN was reliably elicited, no further statistical tests were conducted with SPN amplitude.

#### N200

**FIGURE 1:**
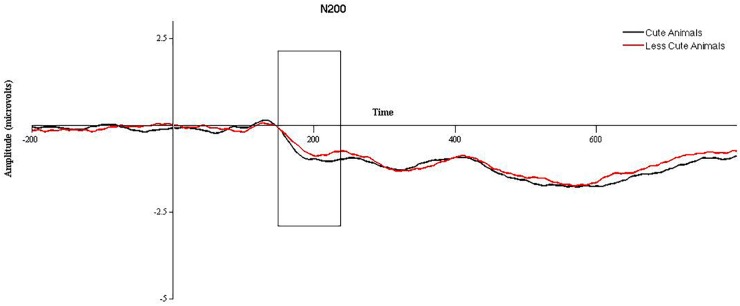
Grand averaged waveforms for the N200 in response to more cute animals (black line) and less cute animals (red line) in anterior electrode sites, collapsed across hemisphere. The area between 150 and 225 ms, used for statistical analysis, is outlined with a black box.

2 (cuteness) × 2 (hemisphere) × 2 (electrode position) repeated measures ANOVAs were run on animal and babies separately. For animals, a significant effect of cuteness was observed, *F*(1,47) = 4.3, *p* = 0.043, such that more cute (baby) animals elicited a larger N200 than less cute (adult) animals, see Figure 1. A significant effect of electrode position was found, *F*(1,47) = 51.08, *p* < 0.001, such that the N200 was significantly larger in anterior versus posterior electrodes. No significant effect of hemisphere was observed, and no significant interactions were found (*p* > 0.05). When the ANOVA was re-run with gender as a between subjects factor, no significant effects of gender were observed. Mediation models in PROCESS for animals were run using amplitude for “more cute” animals in anterior electrode clusters (collapsed across hemisphere) and did not utilize gender as a between subjects factor. For babies, no significant main effects or interactions were observed (*p* > 0.05). When the ANOVA was re-run with gender as a between subjects factor, no significant effects of gender were observed. Therefore, mediation models in PROCESS for babies were run using N200 amplitude for babies (collapsed across “more” and “less” cute conditions, electrode position, and hemisphere) and did not utilize gender as a between subjects factor.

#### Brain and Behavior Correlations

**FIGURE 2:**
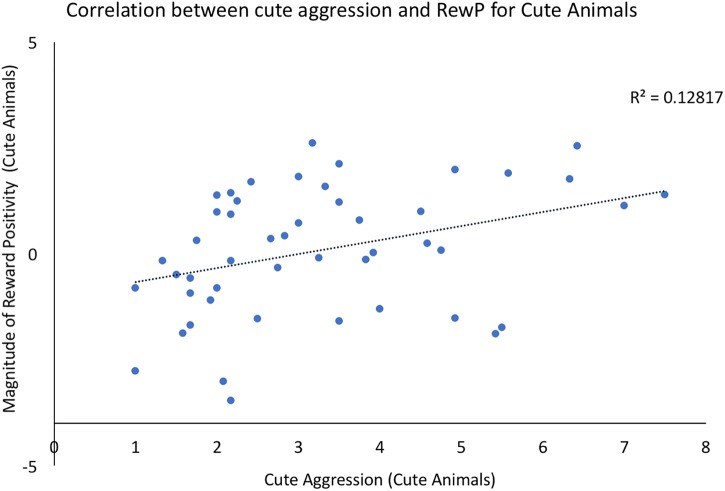
Correlation between behavioral ratings of cute aggression in response to cute animals and RewP amplitude in response to cute animals. Note that RewP amplitude was calculated by subtracting brain activity in the “less cute animals” condition from brain activity in response to the “more cute animals” condition. Therefore, positive RewP amplitude indicates more robust brain activity in the more cute condition compared to the less cute condition.

Bivariate correlations were conducted using the ERP components of interest and behavioral ratings of both cute aggression and dimorphous expression of positive emotions. For each component (N200, RewP), three correlations were run: one between brain activity to cute animals and behavioral ratings of cute aggression in response cute animals, a second between brain activity to babies and behavioral ratings of cute aggression in response to babies, and a third between the ERP component of interest and ratings of dimorphous expressions of positive emotions. Note that because 3 correlations were run for each component, Bonferroni correction lowers the significance threshold to 0.012. Therefore, *p*-values which are less than 0.05 but greater than 0.012 are noted below as “marginally significant,” whereas those under 0.012 are noted as “significant.” A significant correlation was observed between RewP amplitude for cute animals and cute aggression ratings toward cute animals (*p* = 0.012), see Figure 2. A marginally significant correlation was observed between N200 amplitude for cute animals and ratings of dimorphous positive emotions (*p* = 0.044). No other significant correlations were observed.

### Mediation Models

#### Behavior

**FIGURE 3:**
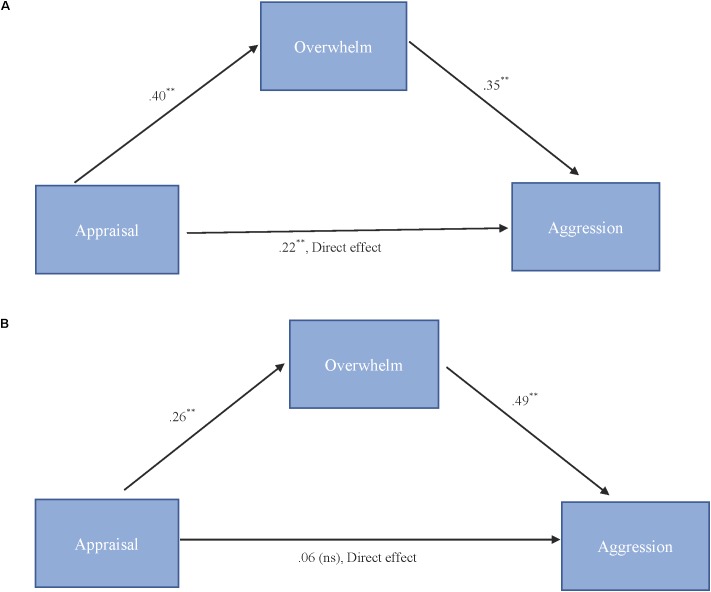
**(A,B)** Behavioral mediation model for “more cute” animals **(A)**. Behavioral mediation model for “more cute” babies **(B)**. **(A)** Model for the relationship between appraisal and cute aggression as mediated by feeling overwhelmed for “more cute” animals. The indirect effect (*b* = 0.14, 95% CI = [0.04, 0.25]) was significant. 5,000 bootstrapped samples, *N* = 47. *^∗∗^p* = 0.01. **(B)** Model for the relationship between appraisal and cute aggression as mediated by feeling overwhelmed for “more cute” babies. The standard coefficient between appraisal and cute aggression when controlling for feeling overwhelmed was not significant. The indirect effect (*b* = 0.13, 95% CI [0.06, 0.23]) was significant. 5,000 bootstrapped samples, *N* = 47. ^∗∗^*p* = 0.01.

As shown in Figure 3A, results for “more cute” animals indicated that appraisal was a significant predictor of feeling overwhelmed, *b* = 0.40, *SE* = 0.11, *p* < 0.001, and that feeling overwhelmed was a significant predictor of expressions of cute aggression, *b* = 0.35, *SE* = 0.10, *p* = 0.001. Approximately 20% of the variance in cute aggression was accounted for by the predictors (*R*^2^ = 0.20). The indirect coefficient was significant, *b* = 0.14, *SE* = 0.05, 95% CI = [0.04, 0.25].

As shown in Figure 3B, results for babies indicated that appraisal was a significant predictor of feeling overwhelmed, *b* = 0.26, *SE* = 0.06, *p* < 0.001, and that feeling overwhelmed was a significant predictor of cute aggression, *b* = 0.49, *SE* = 0.13, *p* < 0.001. Approximately 16% of the variance in ratings of cute aggression was accounted for by the predictors (*R*^2^ = 0.16). The indirect coefficient was significant, *b* = 0.13, *SE* = 0.04, 95% CI = [0.06, 0.23].

**FIGURE 4:**
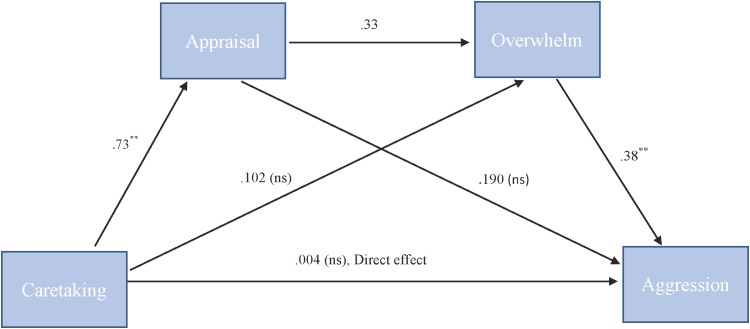
Behavioral serial mediation model for “more cute” animals. Serial mediation model of the effect of caretaking on the outcome of cute aggression, mediated by appraisal and feeling overwhelmed for “more cute” animals. The direct effect of caretaking on cute aggression was not significant. The indirect effect of X and Y was significant (*b* = 0.093, 95% CI = [0.023, 0.211]). 5,000 bootstrapped samples. *N* = 44. ^∗^*p* < 0.05, ^∗∗^*p* < 0.001.

To test our hypothesis that the relationship between caretaking and aggression is serially mediated by both appraisal and feeling overwhelmed we used PROCESS model 6. For “more cute” animals, there was a significant indirect path (*b* = 0.09, 95% CI = [0.02, 0.21]) from caretaking through appraisal (*b* = 0.73, *SE* = 0.08, *p* < 0.001), next through feeling overwhelmed (*b* = 0.33, *SE* = 0.20, *p* = 0.1) to cute aggression (*b* = 0.38, *SE* = 0.10, *p* = 0.001). See Figure 4.

For babies, the serial mediation model was not supported (*b* = 0.04, *ns*). However, for babies, the relationship between feelings of caretaking and cute aggression was mediated by feelings of being overwhelmed (*b* = 0.11). We confirmed simple mediation using Model 4. Results suggested that for babies, caretaking significantly predicted feeling overwhelmed, *b* = 0.22, *SE* = 0.05, *p* < 0.001, and feeling overwhelmed significantly predicted cute aggression, *b* = 0.5, *SE* = 0.14, *p* < 0.001. Approximately 17% of the variance in cute aggression was accounted for by the predictors (*R*^2^ = 0.17). The indirect path from caretaking to cute aggression through feeling overwhelmed was significant, *b* = 0.11, *SE* = 0.03, 95% CI = [0.05, 0.16].

#### Brain and Behavior

##### N200

**FIGURE 5:**
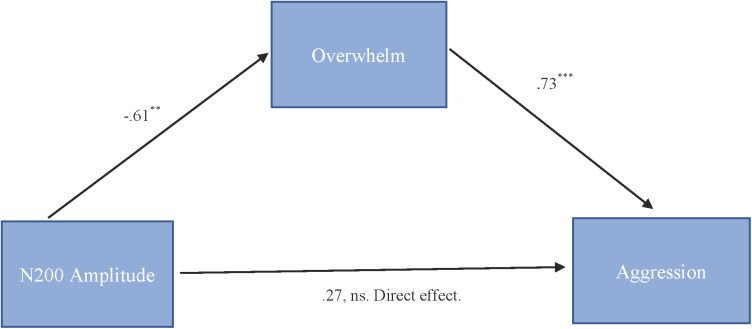
Mediation model of behavior and N200 brain activity in the “more cute” animals condition. Model for the relationship between N200 amplitude and cute aggression as mediated by feeling overwhelmed for “more cute” animals. The relationship between N200 and cute aggression when controlling for feeling overwhelmed was not significant. The indirect effect was significant (*b* = –0.45, 95% CI = [–0.99, –0.14]). 5,000 bootstrapped samples, *N* = 47. *^∗∗^p =* 0.01, ^∗∗∗^*p* < 0.001.

To test our hypothesis that the relationship between N200 amplitude and cute aggression was mediated by feeling overwhelmed, mediation models were run in PROCESS using Model 4. As shown in Figure 5, results for “more cute” animals indicated that N200 amplitude was a significant predictor of feeling overwhelmed, *b* = -0.61, *SE* = 0.23, *p* = 0.01, and feeling overwhelmed was a significant predictor of expressions of cute aggression, *b* = 0.73, *SE* = 0.14, *p* < 0.001. Approximately 0.6% of the variance in cute aggression was accounted for by the predictors (*R*^2^ = 0.006). The indirect coefficient was significant, *b* = -0.45, *SE* = 0.21, 95% CI = [-0.99, -0.14]. For babies, this mediation model was not supported (*b* = -0.33, ns).

##### RewP

**FIGURE 6:**
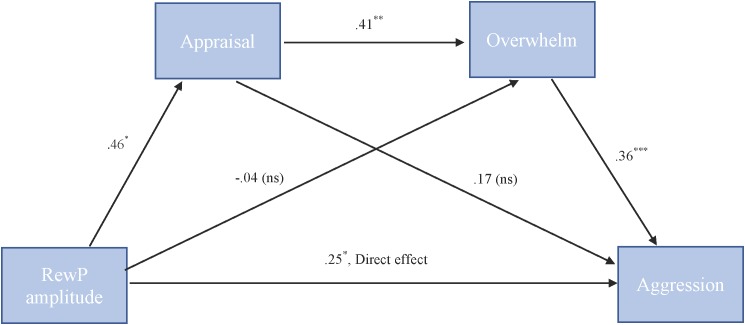
Serial mediation model of behavior and RewP brain activity in the “more cute” animals condition. Serial mediation of the effect of RewP amplitude on the outcome of cute aggression, mediated by appraisal and feeling overwhelmed for “more cute” animals. The direct effect of RewP on cute aggression was significant. The indirect effect of X and Y was also significant (*b* = 0.07, 95% CI = [0.05, 0.18]). 5,000 bootstrapped samples, *N* = 47. ^∗^*p* < 0.05, ^∗∗^*p* < 0.01, ^∗∗∗^*p* < 0.001.

To test our hypothesis that the relationship between RewP amplitude and cute aggression was serially mediated by appraisal and feeling overwhelmed, mediation models were run in PROCESS using Model 6. As shown in Figure 6, for “more cute” animals, there was a significant indirect path (*b* = 0.07, *SE* = 0.04, 95% CI = [0.005, 0.18]) from RewP amplitude through appraisal (*b* = 0.46, *SE* = 0.22, *p* = 0.04), next through feeling overwhelmed (*b* = 0.41, *SE* = 0.11, *p* = 0.001) to cute aggression (*b* = 0.36, *SE* = 0.10, *p* < 0.001). Serial mediation was not supported for babies (*b* = 0.04, *ns*).

**FIGURE 7:**
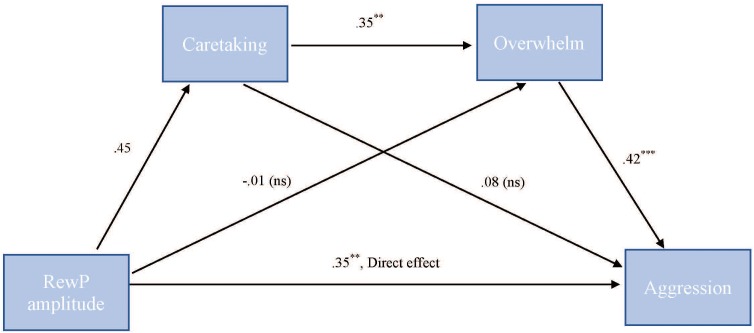
Serial mediation model of behavior and RewP brain activity in the “more cute” animals condition. Serial mediation model of the effect of RewP amplitude on the outcome of cute aggression, mediated by caretaking and feeling overwhelmed for “more cute” animals. The direct effect of RewP on cute aggression was significant. The indirect effect of X and Y was also significant: (*b* = 0.07, 95% CI = [0.003, 0.18]). 5,000 bootstrapped samples. *N* = 44. ^∗^*p* < 0.05, ^∗∗^*p* < 0.01, ^∗∗∗^*p* < 0.001.

To test our hypothesis that the relationship between RewP amplitude and cute aggression was serially mediated by feelings of caretaking and feeling overwhelmed, mediation models were run in PROCESS using Model 6. As shown in Figure 7, for “more cute” animals, there was a significant indirect path (*b* = 0.07, *SE* = 0.04, 95% CI = [0.003, 0.218]) from RewP amplitude through caretaking (*b* = 0.45, *SE* = 0.26, *p* = 0.1), next through feeling overwhelmed (*b* = 0.35, *SE* = 0.11, *p* = 0.003) to cute aggression (*b* = 0.42, *SE* = 0.1, *p* < 0.001). Serial mediation was not supported for babies (*b* = 0.01, *ns*).

## Discussion

The current study was designed to identify and measure neural correlates of cute aggression. “Cute aggression” has been conceptualized as the urge some people get to squeeze, bite, or pinch very cute things without intention to cause harm. Previous findings related to cute aggression suggest these feelings may serve as a mechanism to prevent people from being overwhelmed (and thus incapacitated) by cute things (Aragón et al., 2015).

### Behavioral Findings

Using the same behavioral questionnaires reported by Aragón et al. (2015), we asked participants to rate how much they agreed with statements expressing: cute aggression, feeling overwhelmed, desire to approach, and appraisal of cuteness. Participants filled out these questions after viewing each of four blocks of images: more cute (baby) animals, less cute (adult) animals, more cute babies, less cute babies. Stimuli was the same as reported by Aragón et al. (2015), and images of babies were obtained with permission from Sherman et al. (2013).

Behavioral results for animals were consistent with previous research, as participants gave higher numeric ratings—suggesting higher levels of agreement—to all statements after viewing baby (more cute) versus adult (less cute) animals. Surprisingly, the same pattern was not observed for more versus less cute babies. There were no significant differences in behavioral ratings between the two baby conditions. We hypothesized that this can be explained by task differences between the current study and previous research. Specifically, Aragón et al. (2015) utilized a between subjects’ design in which participants were randomly assigned to either view “more cute” or “less cute” babies. Because we wanted to explore brain activity in response to images, we utilized a within-subjects design in which all subjects viewed images from all conditions. Therefore, our participants viewed both more and less cute babies. This procedural difference may be important for the images of babies because the babies were the same individuals in both conditions–manipulated using Photoshop. That is, participants saw the same 8 babies in both the “more cute” and “less cute” conditions, but the faces were modified to either enhance “cute” features (e.g., larger eyes, fuller cheeks), or to minimize those features (Sherman et al., 2013). Therefore, we hypothesized that participants in the current study were less sensitive to the differences between conditions for babies, which resulted in no observed differences in behavioral ratings. Note that in the animal conditions, photographs were not of the same individual animals, but were images found online depicting either baby or adult animals.

We were interested in how many people in the current study had ever engaged in or heard of behaviors consistent with cute aggression, and how that may relate to their ratings of cute aggression in response to our stimuli. We found positive correlations between ever having squeezed an animal or baby and ratings of cute aggression in response to cute animals and cute babies, respectively. This suggests a relationship between previous behaviors (e.g., squeezing a cute baby or animal) and experiencing cute aggression during the current study. Similarly, we found correlations between people having ever said, “I want to squeeze it!” and ratings of cute aggression to cute animals. Finally, we found correlations between people ever having said, “I want to bite it!” and ratings of cute aggression to cute animals. Taken together, these correlations provide evidence that people who engage in behaviors (either speech or actions) related to cute aggression are more likely to endorse feelings of cute aggression in response to images. These results are encouraging, as they suggest construct validity for the current study.

### Event-Related Potentials

Contrary to our initial hypotheses, the SPN was not elicited by the current paradigm. There are a few potential reasons for this finding: task variability between the current study and previous work, and images not being sufficiently affective in content. Previous studies that have elicited the SPN without a task (e.g., Poli et al., 2007) utilized an S1, S2 paradigm in which the content of S1 informed the participants about the upcoming content in S2. The current study used a block design in which each type of image (e.g., more cute animals, less cute animals) were presented in each block. We did not use an S1, S2 design, because it seemed redundant given the block design. However, it is possible that the lack of an explicit S1 to inform participants about the upcoming content of S2 might explain our inability to measure the SPN. Another potential reason for this finding is due to the nature of the photographs presented. Poli et al. (2007) elicited an enhanced SPN when showing highly affective images (e.g., erotic and gory images). Though our pictures were designed to elicit emotion, it would be unsurprising if these types of images elicit significantly weaker levels of emotion compared to explicit or violent images.

For the N200, we found a main effect of “cute” for cute animals, such that cute animals elicited a significantly larger N200 compared to less cute animals. This is consistent with previous literature relating the N200 to emotion processing (e.g., Streit et al., 2000; Herrmann et al., 2002; Balconi and Pozzoli, 2003), insofar as more cute animals would elicit a larger emotional response than less cute animals. Surprisingly, we did not find analogous results for more versus less cute babies. We interpret this as a less robust difference between the two condition for babies compared to animals. As noted above, different sets of images were used for more and less cute animals (e.g., adult versus baby animals). This was not the case for babies—the same photographs were used in both conditions, but were modified with Photoshop in order to enhance (more cute) or mitigate (less cute) features associated with “cuteness.” Considering that the photographs are of the same babies and that all participants in the current study saw all conditions, it seems plausible that lack of differences in the N200 (and behavioral ratings) can be explained by the two conditions not being sufficiently different for this type of within-subjects design.

### Brain and Behavior Correlations

Prior to running mediation models, we explored the direct relationship between ERP components of interest (N200, RewP) and behavioral ratings of cute aggression. We found a significant correlation between RewP amplitude for cute animals and behavioral ratings of cute aggression toward cute animals. This provides evidence in favor of a relationship between the neural reward system and cute aggression. This is an exciting finding, as it confirms our original hypothesis that the reward system is involved in people’s experiences of cute aggression. Finally, a significant correlation was observed between N200 amplitude for cute animals and individual ratings of dimorphous expressions of positive emotions (e.g., crying when very happy). This is interesting as previous researchers hypothesize that cute aggression is an example of a dimorphous expression and may serve to “regulate” particularly powerful emotions. However, as no relationship was observed between dimorphous expressions of emotion and cute aggression itself, the current study cannot directly speak to that question. In our sample, individuals who had a stronger emotional reaction to cute animals (via the N200), were more likely to report expressing positive emotions with negative expressions (e.g., higher levels of dimorphous expression of positive emotions).

### Mediation Models

Mediation models were utilized to shed light on relationships between multiple variables of interest. In terms of behavior, we found that the relationship between cute aggression and appraisal (e.g., how cute participants found the images) was significantly mediated by feeling overwhelmed. This model was significant for both babies and animals, and is consistent with previous findings (Aragón et al., 2015). Based on the findings of Aragón et al. (2015), we hypothesized that the relationship between caretaking and cute aggression would be serially mediated by appraisal and feeling overwhelmed. Serial mediation was supported for cute animals, but not for cute babies. For cute babies, the relationship between caretaking and cute aggression was significantly mediated by being overwhelmed. These findings are interesting and provide information about how emotional processes occur over time, and how cute aggression may serve to regulate overwhelming emotions. For example, in the case of cute animals, these findings suggest that cute aggression is not simply correlated with caretaking—but is mediated by how cute individuals find animals, and how overwhelmed they feel. This makes sense if one conceptualizes cute aggression as a way to handle overwhelming feelings which occur in response to extremely cute things. As noted by Aragón et al. (2015), it would not be adaptive to be overwhelmed and incapacitated by positive feelings toward cute animals (or babies), if such feelings would interrupt caretaking.

To explore the relationship between behavioral measures and brain activity, mediation models were run with N200 and RewP amplitude. For cute animals, the relationship between N200 amplitude and cute aggression was mediated by feeling overwhelmed. This finding is interesting as it sheds light on how brain activity relates to feelings of cute aggression. As the N200 is hypothesized to be a neural correlate of emotional salience, this suggests that people who find cute animals especially salient and are overwhelmed by those feelings experience cute aggression.

For cute animals, the relationship between RewP amplitude and cute aggression was serially mediated by appraisal and feeling overwhelmed. These findings mirror our behavioral findings (in which feelings of caretaking and cute aggression were mediated by appraisal and feeling overwhelmed). Taken together, these findings suggest that RewP amplitude and feelings of caretaking are similar in their relationship to cute aggression, feeling overwhelmed, and appraisal. Finally, we found that RewP amplitude and cute aggression was serially mediated by feelings of caretaking and feeling overwhelmed. These serial mediation models underscore the complexity of cute aggression, and how it relates to a variety of both neural and behavioral measures (e.g., appraisal, feelings of overwhelm, caretaking, and reward processing). No mediation models were significant for RewP and cute babies.

### Limitations

It is important to discuss limitations of the current study. The most important limitation to consider is the differences in our methods compared to previous research (e.g., Aragón et al., 2015). While Aragón et al. (2015) randomly assigned participants to view either more or less cute animals, the current study used a within-subjects design and showed all images to each participant. The primary goal of the current study was to explore neural correlates of cute aggression, and therefore we decided to directly compare neural responses within each participant. However, it is possible that differences between our methods and those used by Aragón et al. (2015) can account for differences in findings (particularly related to differences between cute animals and babies). A second limitation relates to the stimuli utilized in the current study. In order to be consistent with previous literature on cute aggression (e.g., Aragón et al., 2015), we used the same baby and animal images from Aragón et al. (2015). It is important to note, however, that the animal images were not as controlled as the baby images. That is, images of babies were identical, but manipulated in a photo editing program to make them look more or less “cute” (Sherman et al., 2013). The images of animals, however, were found online and depicted adult animals (less cute) and baby animals (more cute). Thus, images of animals were different on multiple dimensions (e.g., cuteness, age, individual characteristics), whereas images of babies only differed in how prototypically “cute” they were. This is an important consideration, particularly as our findings were most robust for animals, and no differences in brain activity or behavior were observed for images of babies. In addition, analyses on each type of animal (e.g., dog, cat, pig) was not possible due to behavioral questionnaires being asked about each *category* of stimuli (e.g., “more cute animals,” “less cute animals”) rather than individual pictures and inadequate numbers of trials to separate EEG responses by species. As mentioned above, we hypothesized that differences in the current study and Aragón et al. (2015) can be explained by methodological differences. However, we cannot rule out the possibility that the findings in the current study are, in part, explained by stimulus differences between the animal and baby conditions. Future research should employ similar methods of image manipulation in both the baby and animal conditions (as done by Borgi et al., 2014) in order to definitively measure brain activity reflecting cute aggression. Similarly, future research should build upon previous findings that individuals’ feelings toward cute animals are influenced by whether they own pets (Borgi et al., 2014). In the current study, we utilized images of a variety of animal species, including some that are often pets (e.g., cat, dog), and others which are not (e.g., monkey, elephant). Future research related to cute aggression should consider having participants rate different species separately as well as collecting data on whether participants own pets.

Another limitation relates to our study population, and therefore the generalizability of our findings. The current study participants were college students at a large university, rather than a random sample of the general population. It is important to note that college-aged students may have different emotional reactions than individuals in the general population. One example, though anecdotal, underscores these differences. After being debriefed, one participant shared that although she found the babies cute, she felt more “cute aggression” toward the baby animals. She explained that images of cute babies elicited a variety of thoughts related to the future (e.g., ‘Will I have children in the future?’ ‘Do I definitely want children?’ ‘Will I get married?’). She mused aloud that individuals who have children might be more likely to experience cute aggression in response to cute babies compared to those who do not have children. Our participant’s observation aligns with previous research related to brain activities of mothers in response to children’s faces and voices. Previous research in mothers suggest that ERP responses have been associated with parental behavior for interpreting infants’ distress cries (Rutherford et al., 2017) and mental states (Endendijk et al., 2018). In addition, when mothers were given intranasal oxytocin, a hormone involved with social and parental bonding, more robust brain activity was observed for facial expressions (Peltola et al., 2018). We found this idea fascinating and wanted to explore whether participants with children had significant differences in either behavioral or brain measures. Unfortunately, none of our participants had children, so we were unable to perform statistical analyses related to this question. We suggest that future researchers may want to systematically investigate this question.

Another limitation, although not directly related to the current research question, is of note. One participant mentioned that all the babies in our stimulus set were Caucasian. The participant noted that future studies might want to measure cute aggression as it relates to same versus different racial backgrounds (e.g., babies who are of the same versus different race as participants). This in an interesting question that should be explored in future research.

## Conclusion

Overall, our findings suggest that cute aggression is related to neural mechanisms of both emotional salience and reward processing. The current study is the first to our knowledge that explores mechanisms of cute aggression and provides insight into how cute aggression affects brain activity and behavior. Cute aggression appears to be a complex and multi-faceted emotional response that likely serves to mediate strong emotional responses and allow caretaking to occur. It would be of clinical interest and utility to explore whether individuals with disorders related to reward and emotions (e.g., depression, conduct disorders) affects cute aggression, particularly in individuals with conduct disorder who do not experience empathy, or in postpartum mothers who may have difficulty with feelings of caretaking.

## Author Contributions

KS designed the experiments and analyzed the data. LA and KS ran subjects, conceptualized and wrote the paper.

## Conflict of Interest Statement

The authors declare that the research was conducted in the absence of any commercial or financial relationships that could be construed as a potential conflict of interest. The reviewer MB and handling Editor declared their shared affiliation at the time of the review.
